# Protein tyrosine phosphatase PTP4A1 promotes proliferation and epithelial-mesenchymal transition in intrahepatic cholangiocarcinoma via the PI3K/AKT pathway

**DOI:** 10.18632/oncotarget.12116

**Published:** 2016-09-19

**Authors:** Long-Zi Liu, Yi-Zhou He, Ping-Ping Dong, Li-Jie Ma, Zhi-Chao Wang, Xin-Yang Liu, Meng Duan, Liu-Xiao Yang, Jie-Yi Shi, Jian Zhou, Jia Fan, Qiang Gao, Xiao-Ying Wang

**Affiliations:** ^1^ Liver Cancer Institute, Zhongshan Hospital, and Key Laboratory of Carcinogenesis and Cancer Invasion (Ministry of Education), Fudan University, Shanghai 200032, P. R. China; ^2^ Department of Critical Care Medicine, Zhongshan Hospital, Fudan University, Shanghai 200032, P.R, China; ^3^ Department of Gastroenterology and Hepatology, Zhongshan Hospital, Shanghai Institute of Liver Disease, Fudan University, Shanghai 200032, P. R. China; ^4^ Department of Medical Oncology, Fudan University Shanghai Cancer Center, and Department of Oncology, Shanghai Medical College, Fudan University, Shanghai 200032, P. R. China

**Keywords:** PTP4A1, intrahepatic cholangiocarcinoma, prognosis, oncogene, epithelial-mesenchymal transition

## Abstract

The protein tyrosine phosphatase PTP4A1 is a key molecule that activates tyrosine phosphorylation, which is important for cancer progression and metastasis. However, the clinical implications and biological function of PTP4A1 in intrahepatic cholangiocarcinoma (ICC) remains unknown. Here, we showed that PTP4A1 was frequently overexpressed in ICC versus adjacent non-tumor tissues. This overexpression significantly correlated with aggressive tumor characteristics like the presence of lymph node metastasis and advanced tumor stages. Survival analysis further indicated that high PTP4A1 expression was significantly and independently associated with worse survival and increased recurrence in ICC patients. Moreover, through forced overexpression and knock-down of PTPT4A1, we demonstrated that PTP4A1 could significantly promote ICC cells proliferation, colony formation, migration, and invasion *in vitro*, and markedly enhance tumor progression *in vivo*. Mechanistically, PTP4A1 was involved in PI3K/AKT signaling and its downstream molecules, such as phosphorylation level of GSK3β and up-regulation of CyclinD1, in ICC cells to promote proliferation. Importantly, PTP4A1 induced ICC cells invasion was through activating PI3K/AKT signaling controlled epithelial-mesenchymal transition (EMT) process by up-regulating Zeb1 and Snail. Thus, PTP4A1 may serve as a potential oncogene that was a valuable prognostic biomarker and therapeutic target for ICC.

## INTRODUCTION

Intrahepatic cholangiocarcinoma (ICC), originating from the intrahepatic biliary tree, is the second most common primary liver cancer [1, 2]. As one of the most aggressive tumor, clinical outcome for ICC remains dismal in the face of an increasing worldwide incidence [3, 4]. The only curative option for ICC is surgery, but it is limited to patients diagnosed with early-stage disease [5]. Even worse, little progress has achieved in the systematic chemotherapy and molecular target therapies of ICC, which is largely attributed to limited understanding of molecular pathogenesis of ICC [6, 7]. Therefore, grasping the driving events of ICC progression is of paramount importance to identify new effective treatment targets.

The protein tyrosine phosphorylation level is regulated by both protein tyrosine kinases (PTK) and phosphatases (PTP) [8]. Similar to the PTKs, dys-regulation of protein phosphatases has been linked to tumor onset and progression [9]. Recently, Tai et al discovered that activating the tumor suppressive phosphatase SHP-1 could result in better anti-HCC effect than sorafenib, highlighting the therapeutic value of targeting PTPs [10]. Moreover, our previous study using whole-exome sequencing in 7 ICCs showed that 9 PTPs were mutated in 4 out of 7 ICCs and prevalence of mutations of ICCs in at least one PTP was 51.3% (64/124), which is much higher than TP53 mutation prevalence (31.5%, 39/124) [11]. These findings further highlighted the importance of PTPs in ICC carcinogenesis. However, in sharp contrast to great success of target therapy in PTKs, target therapy in PTPs has limited progression, especially for ICC. Therefore, detailed investigations on PTPs in ICC will provide promising targets for ICC treatment.

PTP4A1 was first identified and isolated as a novel PTP in mitogen-stimulated cells and regenerating liver [12]. Accumulating evidence showed that PTP4A1 plays a stimulative role in various pathological processes, especially in carcinogenesis. Overexpression of PTP4A1 in non-tumorigenic normal cells leads to a transformed phenotype, rapid proliferation [13] and clonogenic growth in soft agar [14]. In addition, PTP4A1 overexpressed cells exhibit elevated migration and invasion abilities, and are capable of forming metastatic tumors in nude mice whereas corresponding controls cannot [15, 16]. Moreover, down-regulation of endogenous PTP4A1 expression in cancer cells results in reduced proliferation and suppressed cell motility [17]. Mechanically, PTP4A1is involved in different downstream signaling pathways in different types of cancer, including Rho GTPases family in colorectal carcinoma [16], c-Src level [17] and focal adhesion components (like p130Cas) [18] in lung cancer. The function and mechanism of PTP4A1 in HCC has been revealed by a recent study indicating that PTP4A1 represses E-cadherin through activating PI3K/AKT signaling pathway [19]. However, the role of PTP4A1 in ICC remains largely unknown. In this study, we explored the clinical relevance and biological significance of a potential oncogene PTP4A1 in human ICC.

## RESULTS

### Expression pattern of PTP4A1 in ICC

**Figure 1:**
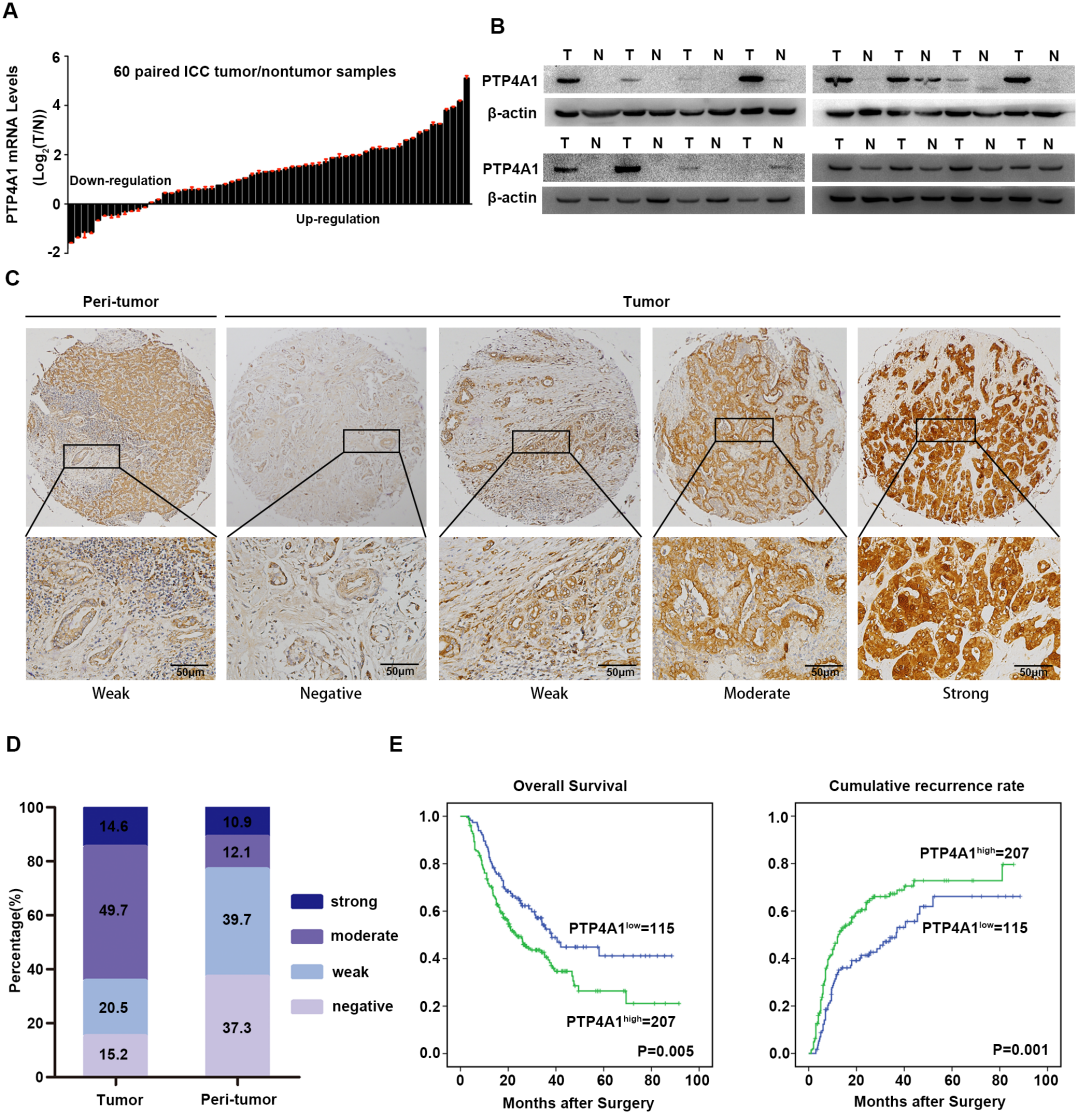
Expression pattern and clinical implications of PTP4A1 in ICC **A.** PTP4A1 mRNA expression in 60 paired ICC tumor and adjacent non-tumor tissues. **B.** The protein expression of PTP4A1 in tumor tissues (T) versus adjacent non-tumor tissues (N) (n=16). **C.** Representative immunostaining pictures of PTP4A1 in ICC tumor and adjacent non-tumor tissues. Left: adjacent non-tumor tissues and right: different staining intensities in ICC tumors. Scale bars =100μm. **D.** Bar graph showed the percentage of different staining intensity for PTP4A1 in 322 ICC patients. **E.** Kaplan-Meier curves for overall survival (OS) and time to recurrence (TTR) based on PTP4A1 expression in ICC cohort (n=322).

To investigate the potential role of PTP4A1 in ICC, mRNA expression was first evaluated in 60-paired ICC tumor and adjacent non-tumor tissues. The results showed that overexpression of PTP4A1 were observed in 80% (48/60) of ICC patients (Figure 1A). Then, in randomly selected 16-paired ICC tumor and adjacent non-tumor tissues, western blot analysis showed obviously elevated protein expression of PTP4A1in ICC (Figure 1B). Immunohistochemically, PTP4A1 staining intensity was weak in adjacent normal intrahepatic biliary tissues, as compared with marked up-regulation in ICC tumor tissues (Figure 1C). PTP4A1 was scored as strong or moderate intensity in 64.3% (207 of 322: strong, n=47; moderate, n=160) of tumor tissues, whereas only 23.0% (74 of 322: strong, n=35; moderate, n=39) of corresponding adjacent normal intrahepatic biliary tissues was scored as strong or moderate intensity (Figure 1D). Our results showed that PTP4A1 mRNA and protein were frequently overexpressed in human ICC.

### PTP4A1 overexpression is associated with aggressive clinicopathological characteristics and poor prognosis

We then focused on the clinical implications of PTP4A1 expression in ICC patients. Based on PTP4A1 immunostaining intensity, the cohort of 322 ICC patients was divided into high (strong and moderate intensity) and low (weak and negative intensity) groups. High PTP4A1 expression significantly correlated with aggressive tumor characteristics, including larger tumor size (*P* = 0.020), lymph node metastasis (*P* = 0.002) and advanced tumor stage (*P* = 0.004) (Supplementary Table 3). Moreover, high expression of PTP4A1 was significantly associated with decreased survival and increased risk for postoperative recurrence in ICC patients (Figure 1E). Patients in high PTP4A1 expression group had obviously worse overall survival (OS) (median OS 23.0 and 38.2 months respectively; difference=15.2 months; *P*=0.005) and shorter time to recurrence (TTR) (median TTR 12.3 and 36.7 months respectively; difference=24.4 months; P=0.001) than those in low expression group. As summarized in Supplementary Table 4, multivariate analysis revealed that PTP4A1 expression remained to be an independent prognostic factor for both OS (hazard ratio=1.479, 95% CI 1.063-2.056, *P*=0.020) and TTR (hazard ratio=1.587, 95% CI 1.149-2.192, P=0.005). Collectively, these data suggested that high expression of PTP4A1 was a valuable index for dismal prognosis in ICC patients.

### PTP4A1 promotes ICC cells proliferation and invasion *in vitro*

**Figure 2:**
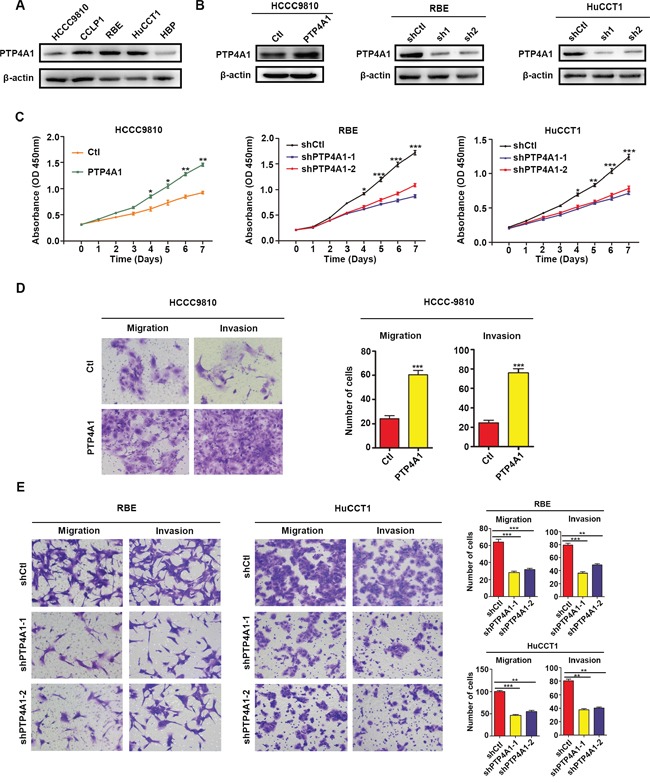
PTP4A1 promotes ICC cell proliferation, colony formation and invasion *in vitro* **A.** Relative PTP4A1 protein level in cell lines. β-actin was used as a loading control. **B.** Efficiency of PTP4A1 overexpression and down-regulation in ICC cells. **C.** Effects of PTP4A1 overexpression and down-regulation on proliferation. **D.** Effects of PTP4A1 overexpression on migration and invasion using transwell assay. Representative images were shown. Magnification: ×200. **E.** Effects of PTP4A1 down-regulation on migration and invasion using transwell assay. Representative images were shown. Magnification: ×200. All bar graphs depicted quantification of triplicate results with mean ± SD. *P<0.05, **P<0.01, ***P<0.001.

The clinical implications of PTP4A1 in ICC promoted us to explore its potential biological function. Constitutive expression of PTP4A1 in 4 ICC cell lines, as well as a normal biliary epithelial cell HBP, were confirmed (Figure 2A). HCCC9810, a cell line with low level of PTP4A1, was transfected with PTP4A1-overexpression lentivirus and its control. RBE and HuCCT1 cell lines, which expressed high endogenous PTP4A1, were transfected with PTP4A1-shRNA lentivirus and their control, respectively. PTP4A1 overexpression and knockdown efficiency were confirmed using western blot (Figure 2B). The *in vitro* proliferation of PTP4A1 overexpressed ICC cells was significantly increased compared to control cells from day 4. Likewise, in PTP4A1 silenced ICC cells, significantly decreased proliferation was detected from day 4 compared to control cells (Figure 2C). Significantly elevated colony formation was observed in HCCC9810-PTP4A1 ICC cells, while reduced colony formation was detected in PTP4A1 silenced ICC cells (Supplementary Figure 1B). PTP4A1 overexpressed ICC cells showed significantly enhanced cell motility and invasion compared to control cells as measured by scratch (Supplementary Figure 2A), migration and invasion assays (Figure 2D). In contrast, migration and invasion of PTP4A1 silenced cells were evidently inhibited compared to control cells (Supplementary Figure 2B and Figure 2E). Considering that no significant difference in cell number was detected within 3 days, the cell motility change should not be attributed to cell proliferation using these assays within 3 days. Taken together, PTP4A1could promote the proliferation, colony formation and invasion of ICC cells *in vitro*.

### PTP4A1 promotes ICC progression *in vivo*

**Figure 3:**
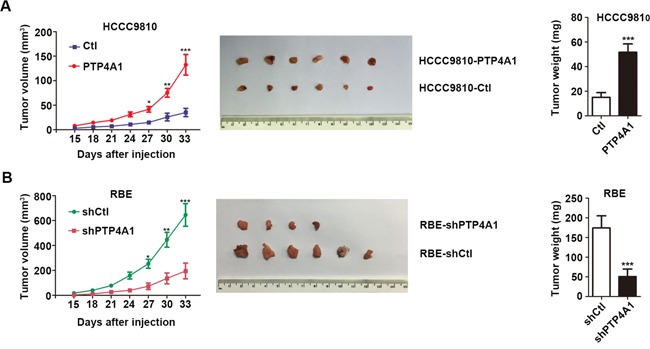
PTP4A1 promotes ICC progression *in vivo* **A.** Effects of PTP4A1 overexpression on *in vivo* subcutaneous xenograft tumors. Tumor volume and weight increased significantly between HCCC9810-PTP4A1 and HCCC9810-Ctl group (n=6). **B.** Effects of PTP4A1 down-regulation on *in vivo* subcutaneous xenograft tumors. All the mice successfully formed palpable tumors, except two mice in RBE-shPTP4A1-1 group. Tumor volume and weight increased markedly between RBE-shCtl and RBE-shPTP4A1-1 group (n=6). *P<0.05, **P<0.01, ***P<0.001.

Next, we performed subcutaneous xenograft tumor models using HCCC9810-PTP4A1 cells, RBE-shPTP4A1-1 cells, and their relative controls. Two weeks after injection, all mice successfully formed palpable tumors, except two mice in RBE-shPTP4A1-1 group. The tumor growth curves showed that tumors in HCCC9810-PTP4A1 and RBE-shCtl groups grew faster than in HCCC9810-Ctl and RBE-shPTP4A1-1 groups at the same time period, respectively. Tumor volume of HCCC9810-PTP4A1-derived and RBE-shCtl-derived xenografts was 132.34 ± 51.32 mm^3^ and 645.34 ± 223.43 mm^3^, respectively, significantly larger than that derived from HCCC9810-Ctl and RBE-shPTP4A1-1 groups (35.15 ± 20.85 mm^3^ and 195.26 ± 154.45 mm^3^, respectively, *P*<0.001). Similarly, the weight of tumors derived from HCCC9810-PTP4A1 and RBE-shCtl groups was evidently heavier than HCCC9810-Ctl and RBE-shPTP4A1-1 groups (Figure 3A and 3B). Collectively, these results indicated that PTP4A1 could significantly promote ICC growth and progression *in vivo*.

### PTP4A1 regulates ICC cell proliferation and invasion via activating PI3K/AKT signaling pathway and EMT

**Figure 4:**
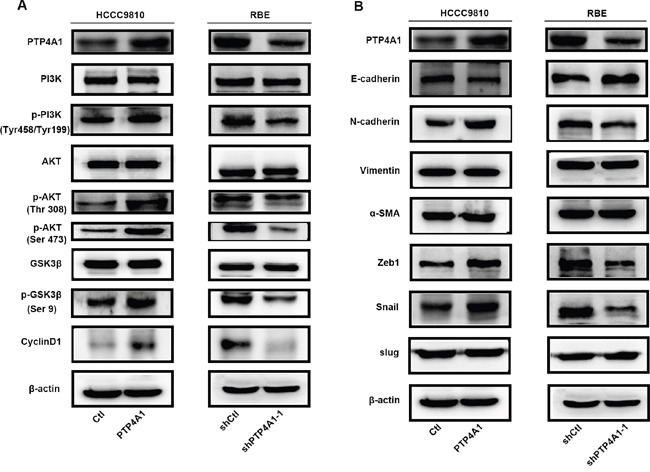
PTP4A1 regulates ICC cell proliferation and invasion via activating PI3K/AKT signaling pathway and EMT **A.** Protein levels of PI3K, p-PI3K (Tyr199), AKT, p-AKT (Thr308), p-AKT (Ser473), GSK3β, p-GSK3β (Ser9), CyclinD1 were shown in indicated cells. **B.** Protein levels of epithelial and mesenchymal makers, and transcription factors were compared in indicated cells. β-actin was used as loading control.

In consideration of previous reports indicated that PTP4A1 repressed E-cadherin through PI3K/AKT signaling pathway [19], we wondered whether PTP4A1 similarly activated PI3K/AKT signaling in ICC cells. Consistently, overexpression of PTP4A1 in HCCC-9810 cells caused a significant increase in AKT (Thr308, Ser473) and GSK3β (Ser9) phosphorylation levels, as compared with control cells. In contrast, phosphorylation of AKT and GSK3β were suppressed in PTP4A1-shRNA lentivirus-transfected RBE cells. Interestingly, cell-cycle regulator CyclinD1 was obviously enhanced in HCCC9810-PTP4A1 cells and down-regulation of CyclinD1 was detected in RBE-shPTP4A1-1 cells as compared with their respective controls (Figure 4A). These results suggested that activation of PI3K/AKT signaling pathway and downstream molecules, such as GSK3β and CyclinD1, by PTP4A1 in ICC cells may be the underlying mechanism for its proliferation-promoting effect.

Epithelial-mesenchymal transition (EMT) is one main process that provides cancer cells with increased migratory and invasive abilities [20]. Therefore, key molecules of EMT were evaluated in both PTP4A1 overexpressed and silenced ICC cells. Increased E-cadherin expression and down-regulation of N-cadherin were observed in PTP4A1 silenced ICC cells. Conversely, down-regulation of E-cadherin and up-regulation of N- cadherin were detected after PTP4A1 was overexpressed in ICC cells. However, expression of Vimentin and α-SMA remained to be similar regardless of PTP4A1 expression (Figure 4B).

Interestingly, Zeb1 and Snail expression, rather than Slug, were significantly and negatively associated with expression of E-cadherin. Zeb1 and Snail expression levels were decreased in PTP4A1 silenced ICC cells, while up-regulation of Zeb1 and Snail were detected in PTP4A1 overexpressed ICC cells (Figure 4B). Taken together, our results suggested that PTP4A1 may regulate EMT of ICC cells through two transcriptional factors Zeb1 and Snail.

**Figure 5:**
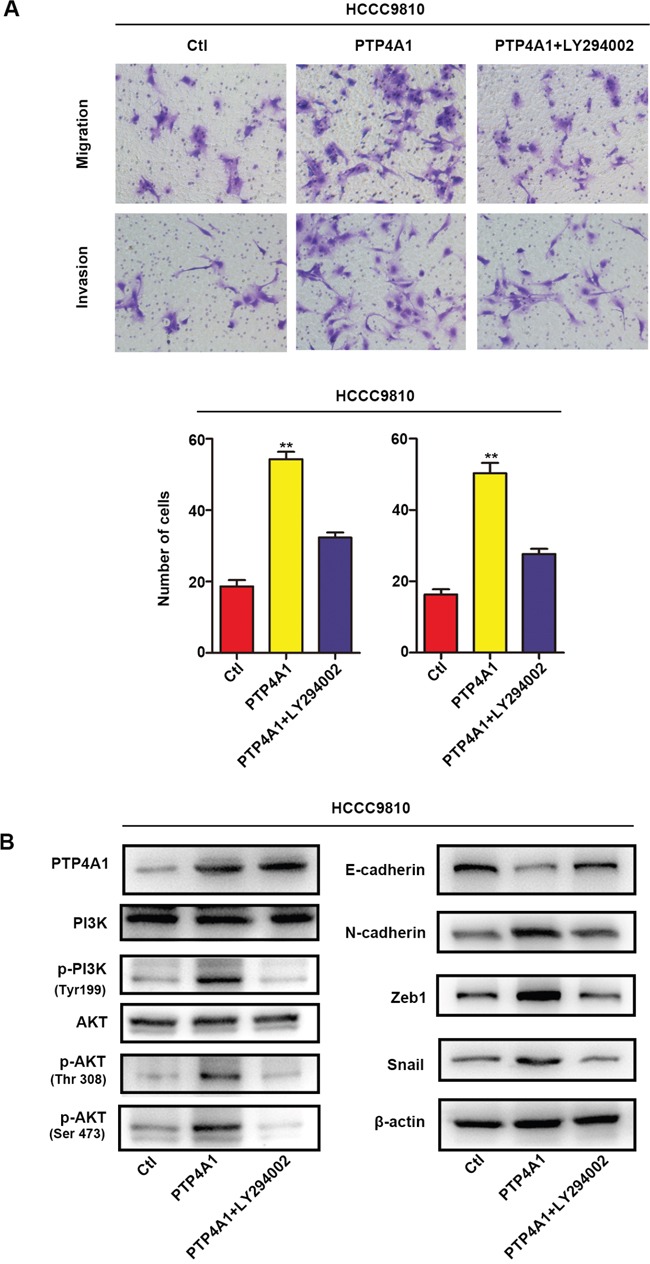
Overexpression of PTP4A1 promotes EMT process through PI3K/AKT signaling pathway **A.** Stable HCCC-9810 cells were treated with or without the PI3K inhibitor LY294002, and applied to migration and invasion assays. Representative images were shown in each group. Magnification: ×200. All bar graphs depicted quantification of triplicate results with mean ± SD. *P<0.05, **P<0.01, ***P<0.001. **B.** Protein levels of PI3K, p-PI3K (Tyr199), AKT, p-AKT (Thr308), p-AKT (Ser473), epithelial and mesenchymal makers, and transcription factors were compared in indicated cells. β-actin was used as loading control.

Previous articles reported that PI3K/AKT signaling pathway played a crucial role in EMT process through a variety of ways to enhance tumor cells metastasis and aggressiveness [21, 22]. In line with this, we found that PI3K inhibitor LY294002 could markedly decreased migration and invasion of PTP4A1 overexpressed ICC cells *in vitro* (Figure 5A). In addition, LY294002 also caused the down-regulation of N-cadherin, Zeb1 and Snail, as well as the up-regulation of E-cadherin in PTP4A1-overexpression lentivirus transfected HCCC-9810 cells (Figure 5B). These results implied that PTP4A1 promotes EMT in ICC mainly through the PI3K/AKT signaling pathway.

## DISCUSSION

Accumulative data have revealed that aberrant phospho-tyrosine signaling cascades regulated by both tyrosine kinases and phosphatases could significantly influence cell growth, migration and metastasis in various kinds of tumors [23, 24]. Although PTP4A1 has been investigated in different pathological processes, this study for the first time demonstrated a potential oncogenic role of PTP4A1 in ICC.

Several lines of evidence support the hypothesis that PTP4A1 may serve as an oncogene in ICC. First, PTP4A1 was significantly overexpressed and served as an independent prognosticator for clinical outcome in ICC. Results from previous studies of PTP4A1 in ovarian carcinoma [25] and HCC [19] regarding its clinical implications were consistent with our findings. In addition, the relatively large sample size of our ICC cohort further solidified our findings, considering ICC is a relatively rare liver malignancy and few series had more than 100 patients from a single center. Second, down-regulation of PTP4A1 significantly inhibited, while overexpression of PTP4A1 significantly promoted cell proliferation, colony formation, migration, and invasion of ICC cells *in vitro*. These results were highly in line with previous studies in human embryonic kidney cells [13], in which the growth-stimulating effect of PTP4A1 was reported. Meanwhile, the invasion-promoting effect of PTP4A1 was also revealed in colorectal cancer [16], lung cancer [17] and HCC [19]. Third, we demonstrated that PTP4A1 promoted cell proliferation via PI3K/AKT signaling pathway and downstream molecules, such as GSK3β and CyclinD1, consistently with a recent report that activation of PI3K/AKT signaling in beta cells may promote beta-cell proliferation through increased cellular level of cell-cycle regulator CyclinD1 [26].

The mechanisms for invasion-promoting effect of PTP4A1 in different types of cancers are highly different. Previous studies indicated that PTP4A1 promoted cell migration and invasion by regulating filamentous action dynamics [27] and matrix metalloproteinase expression [28]. Rho family GTPases were responsible for increased cell motility in PTP4A1 overexpressed colorectal cancer [16] and c-Src expression played an important role in PTP4A1 regulated lung cancer [17], whereas PTP4A1-mediated E-cadherin expression through PI3K/AKT signaling pathway was crucial to HCC motility [19]. Therefore, the identification of underlying mechanism of invasion-promoting effect of PTP4A1 in ICC needs a cancer-specific exploration. As EMT is strongly hypothesized as a driver of cancer cell motility and ICC shares similar oncogenic processes with HCC, we explore and indeed found that E-cadherin was also regulated by PTP4A1 in ICC cells. We demonstrated that overexpression of PTP4A1 could activated PI3K/AKT signaling and positively regulate EMT process. Furthermore, two transcriptional factors Zeb1 and Snail were identified to be involved in PTP4A1 regulated EMT process. Consistently, a recent study indicated that Zeb1 expression was positively accordant with tumor development and poor prognosis in ICC patients [29]. However, the detailed mechanism attributed to PTP4A1 mediated EMT process in ICC need further exploration.

In summary, our study demonstrated that PTP4A1 was overexpressed in ICC and played an important role in the progression and metastasis of ICC. The functional role of PTP4A1 was assumed to be acted by activation PI3K/AKT signaling and promoting the EMT process through two pivotal transcriptional factors Zeb1 and Snail. The clinical relevance and functional significance of PTP4A1 in ICC make it a promising therapeutic target for future drug development.

## MATERIALS AND METHODS

### Cell lines

RBE cell line was purchased from Cell Resource Center of Tohoku University (Tohoku, Japan), and HCCC-9810, HuCCT1, CCLP1, as well as a normal biliary epithelial cell line (HBP) were purchased from Chinese Academy of Sciences Shanghai Branch Cell Bank (Shanghai, China). Cell lines were cultured in RPMI Medium 1640 (Gibco, USA) supplemented with 10% fetal calf serum (Gibco, USA), penicillin (100 unites/ml) and streptomycin (100 ug/ml) at 37°C in a thermostatic incubator with 5% CO_2_.

### Patients and specimens

A total of 322 paraffin-embedded ICC tumor and matched adjacent non-tumor tissues were collected from our department (Liver Cancer Institute, Zhongshan Hospital of Fudan University) between 2005 and 2011 [11, 30]. Overall survival and time to recurrence was defined as a time frame from the date of operations to death and recurrence, respectively. If recurrence was not appeared or patients were still alive at the last follow-up, data were censored. 60 pairs of fresh ICC specimens and matched adjacent non-tumor tissues were obtained for quantitative reverse transcription PCR analysis, and 16 pairs were randomly selected for western blot analysis. The written informed consent from patients and the Ethical approval form Zhongshan Hospital Research Ethics Committee were obtained.

### Tissue microarray and immunohistochemistry

Procedures and details of tissue microarray construction were as previously described [31]. Immunohistochemistry for PTP4A1 was performed using a two-step protocol as previously described [32]. Briefly, paraffin sections were baked for 30min at 70°C, de-paraffinized in xylene, rehydrated in gradually varied alcohol, and then the sections were managed with 1% H_2_O_2_ to neutralize endogenous peroxidase for 30min. The antigen retrieval was processed with incitrate buffer (pH=6.0) in a microwave oven. After antigen retrieval, the sections were incubated with primary antibody PTP4A1 and secondary antibody. The sections were then stained with DAB (3,3-diaminobenzidine) and terminated in PBS, and then counterstained with hematoxylin. Based on the staining intensity of PTP4A1 in each case, the standard for score was as follows: 0, negative; 1, weak; 2, moderate; 3, strong. Two observers graded the score of staining intensity independently.

### Proliferation, colony formation and wound healing assays

For proliferation assay, cholangiocarcinoma cells were planted in 96-well plates at the amount of 1000 cells/well and observed a week according to the growth characteristics. Tumor cell count was calculated via OD value at 450 nm on each day by Cell-Counting Kit (CCK8; Dojindo, Japan) according to the manufacturer's instructions.

For colony formation assay, cells were planted in 6-well plates at the mount of 500-1000 cells/well, and observed two weeks according to the size of cell colony. Subsequently, the cells were fixed in 4% paraformaldehyde for 30min and then stained with crystal violet staining solution for 30min.

For wound healing assay, cells were planted in 6-well plates and waited to cover 90% of plate bottom, scraped by a bio-clean tip of a 200μl pipette to generate the constant width of an artificial “wound”. Then, the gap (wound) was photographed at 0h, 24h and 36h, respectively. The ability of cell migration movement was evaluated by measuring the change of “wound” width.

All results were representative of three independent experiments performed in triplicate.

### Migration and invasion assays

The migration and invasion assays were carried out in chamber of 8-μm pore size (Corning, USA) as previous described [11]. For migration assay, 1 × 10^4^ tumor cells in 200μl DMED without FBS were added in the upper chamber, while 500μl DMEM with 30% FBS was placed in the lower chamber. The chambers were placed in 24-well plates, and incubated in a thermostatic incubator with 5% CO_2_ at 37°C for 48h. Subsequently, cells were fixed in 4% paraformaldehyde for 30min and then stained with crystal violet staining solution for 30min. As to invasion assay, 5 × 10^5^ tumor cells were used, and each chamber should be pre-coated with 50μl BD Matrigel (diluted 1:5 with RPMI Medium 1640) in 37°C for 3 hours in advance. The following procedures of the invasion assay were the same with migration assay. Cells in 5 random fields were counted using an IX71 inverted microscope (Olympus Corp) and then the mean value was recorded.

### Tumor models

Four-week-old male NOD/SCID nude mice were purchased from Shanghai SLAC Laboratory Animal Co., Ltd (Shanghai, China) and raised according to the guidelines provided by Shanghai Medical Experimental Animal Care Commission [33]. Cell suspensions (5 × 10^6^ cells) were subcutaneously injected into the flank of each mouse. Tumor volume was measured every 3 days and calculated with the following formula: 0.5 x length x width^2^. At the end of experiment, mice were sacrificed and tumors were collected, weighted, and photographed.

### RNA isolation and quantitative reverse transcription PCR (qRT-PCR)

The total RNA was isolated with TRIzol reagent (Invitrogen, USA) and reverse-transcribed to cDNA using PrimeScript RT reagent kit (Takara, Japan). For real-time RT-PCR, SYBR Premix Ex Taq™ (Takara, Japan) was used according to the manufacturer's instructions as previously described [32]. Primers were designed as follows: PTP4A1, forward: 5′-ACCAATGCGACCTTAAACAAA-3′and reverse: 5′-AATCTGGTTGGATGGTGGTG-3′; GAPDH, forward: 5′-AGCCACATCGCTCAGACAC-3′and reverse: 5′-GAATTTGCCATGGGTGGA-3′. The DNA amplification and detection were accomplished by applying the ABI PRISM 7900 Sequence Detection System (Applied Biosystems, USA). Triplicate qRT-PCR samples were performed in each assay.

### Lentivirus construction and transfection

The lentiviral packaging system was composed of the vectors pGCL-puro, pHelper 1.0 and pHelper 2.0, provided by Genechem (Shanghai, China). Three plasmids (pHelper 1.0, pHelper 2.0, and pGCL-puro or pGCL-puro-PTP4A1) were co-transfected into 293T cells using Lipofectamine 2000 (Invitrogen, USA). After 12 hours, the cell culture medium was replaced with fresh medium containing 10% fetal calf serum. The lentivirus supernatant containing PTP4A1 cDNA was collected at 48 hours after co-transfection. Lentivirus supernatant was transfected into HCCC-9810 cells with 8μg/mL polybrene (Sigma-Aldrich, USA) to obtain stable cell line, named HCCC9810-PTP4A1. The HCCC9810-Ctl was used as a control.

A set of shRNA (short hairpin RNA) lentiviral vectors differed in PTP4A1-targeting sequences (Supplementary Table 1) and pLKO.1-puro empty vector with green fluorescent protein (GFP) were purchased from Genechem (Shanghai, China). The lentiviral packaging system was the same as mentioned above. Lentivirus supernatant was transfected into RBE cells with 8μg/mL polybrene (Sigma-Aldrich, USA) to obtain stable cell line, named RBE-shPTP4A1-1 and RBE-shPTP4A1-2. The RBE-shCtl was used as a control.

Approximately 48 hours after lentivirus transfection, 2μg/ml puromycin was used for 1 week. The efficiency of overexpression and knockdown were validated by western blot.

### Western blot

Total protein from HCC cells and tumor specimens were extracted and separated in 10% SDS-PAGE, and then electro-transferred onto PVDF membranes. After blocked in Blocking Buffer (Beyotime, China) for 1 hour, the membranes were incubated with primary antibody PTP4A1 and secondary antibody according to protocols of the manufacturer. Primary antibodies were listed in Supplementary Table 2.

### Statistical analysis

Statistical analyses were conducted using SPSS 18.0 software. Analysis of PTP4A1 expression with clinicopathologic parameters were performed using Pearson Chi-square test. Variables associated with OS and TTR was identified using univariate Cox proportional hazards regression models. Significant factors in univariate analysis were further subjected to a multivariate Cox regression analysis in a stepwise manner. Kaplan-Meier plots (log-rank tests) were used to describe OS and TTR. The results of functional experiments were presented as the means ± standard deviation and evaluated using a Mann–Whitney *U* test. A two-tailed *P*<0.05 was considered significant.

## SUPPLEMENTARY FIGURES AND TABLES


