# Supplementary material for: Protein tyrosine phosphatase PTP4A1 promotes proliferation and epithelial-mesenchymal transition in intrahepatic cholangiocarcinoma via the PI3K/AKT pathway
Source: Oncotarget. 2016 Sep 19;7(46):75210–20. doi: 10.18632/oncotarget.12116 (PMC5342735; doi:10.18632/oncotarget.12116)
Supplement: Supplementary file 1 [file oncotarget-07-75210-s001.pdf]

# Protein tyrosine phosphatase PTP4A1 promotes proliferation and epithelial-mesenchymal transition in intrahepatic cholangiocarcinoma via the PI3K/AKT pathway

## SUPPLEMENTARY FIGURES AND TABLES

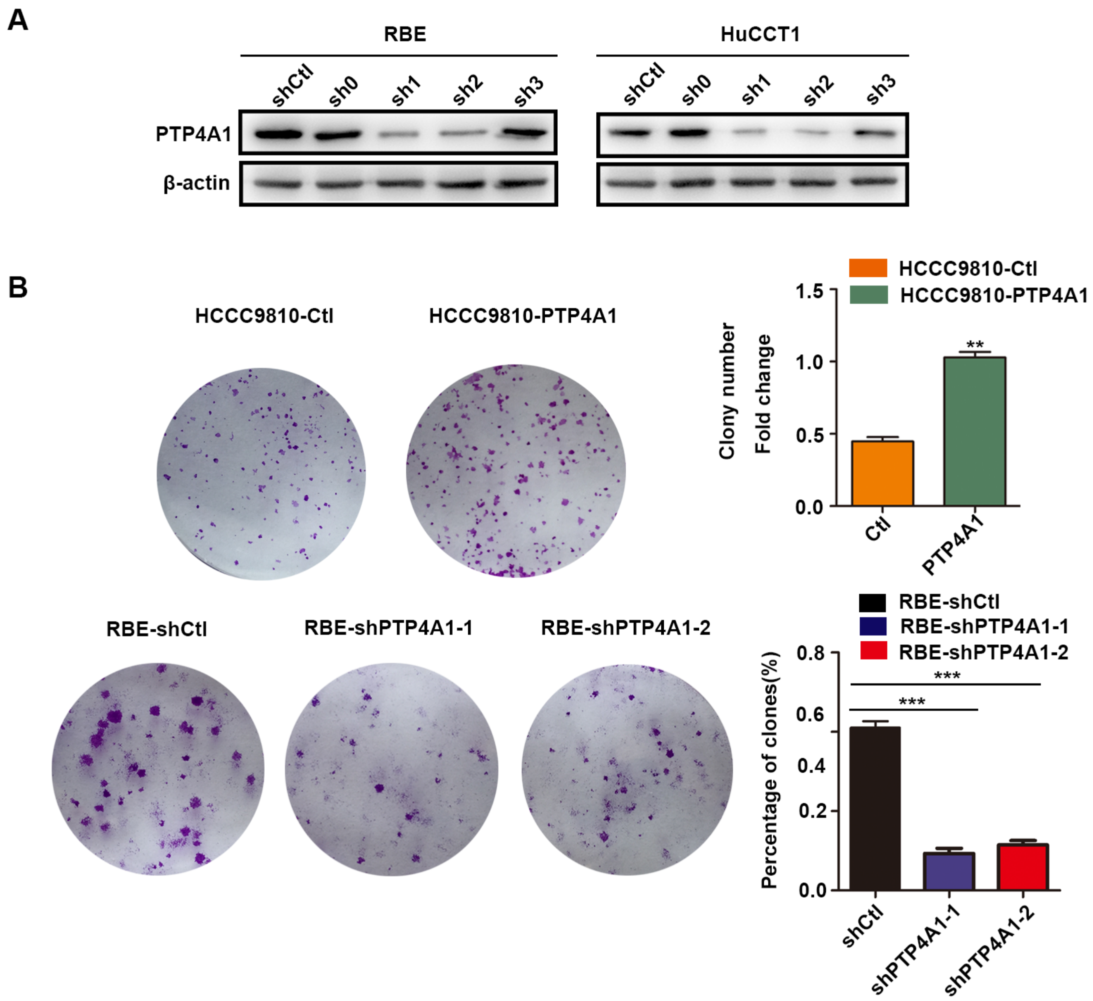


**Supplementary Figure S1: Knockdown efficiency of PTP4A1-shRNA lentivirus and colony formation capacity of PTP4A1 overexpression and down-regulation ICC cells. A.** PTP4A1 knockdown efficiency of 4 RBE-shPTP4A1 vectors and control vector in RBE and HuCCT1 cells were confirmed using western blot. **B.** Effects of PTP4A1 overexpression and down-regulation on proliferation were confirmed using colony formation assay. Representative images of colony formation were shown (left panel). All bar graphs depicted quantification of triplicate results with mean ± SD. *P<0.05, **P<0.01, ***P<0.001.

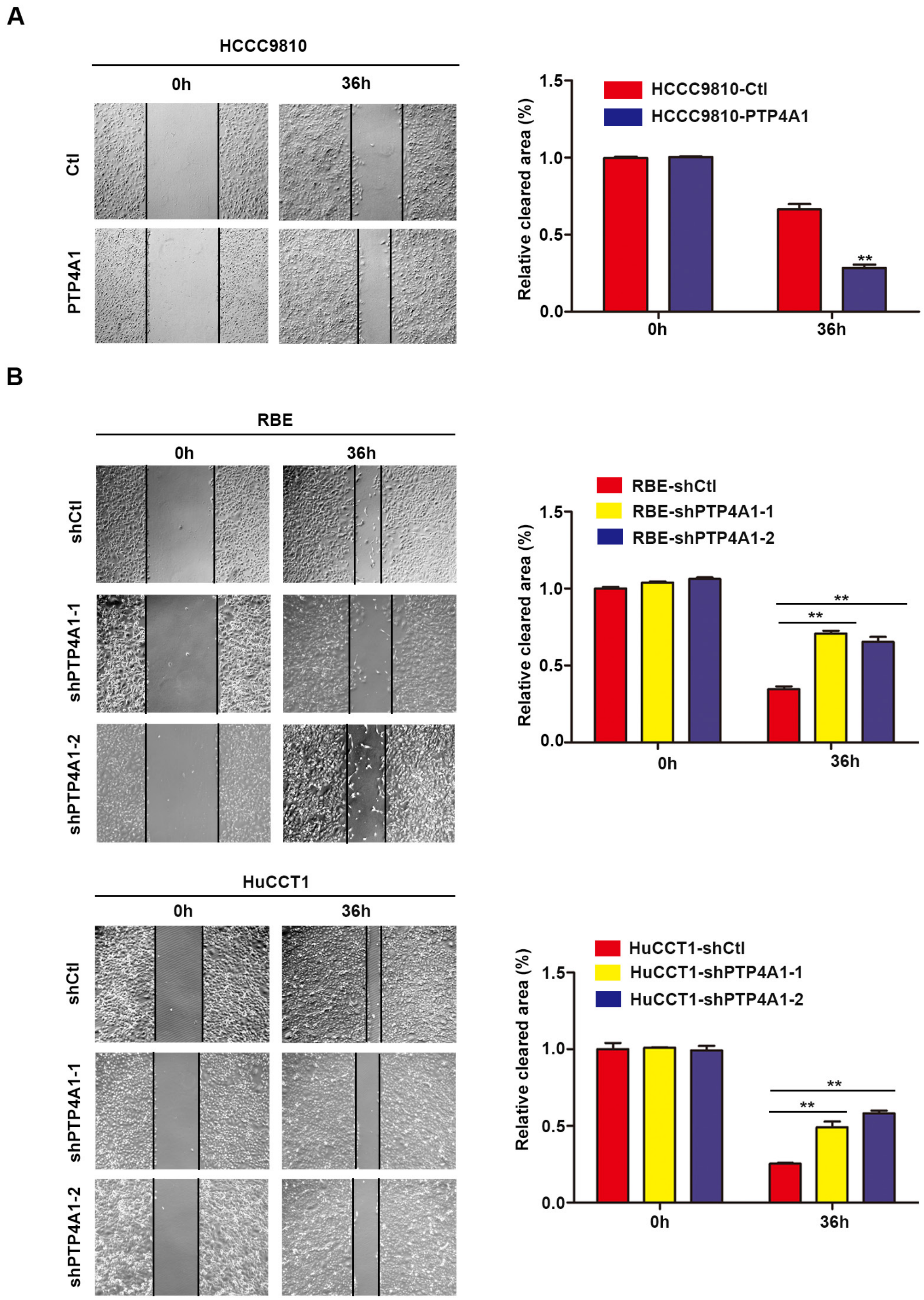


**Supplementary Figure S2: Effects of PTP4A1 overexpression and down-regulation on migration using scratch assay.** **A-B.** Representative images of colony formation were shown (left panel). All bar graphs depicted quantification of triplicate results with mean ± SD. *P<0.05, **P<0.01, ***P<0.001.

**Supplementary Table S1: Target sequences of shPTP4A1**

| Gene | List | Target sequence |
|---|---|---|
| PTP4A1 | sh0 | CTTAAGAAGTATGGAGTTACC |
| | sh1 | CCAACCAATGCGACCTTAAAC |
| | sh2 | CTGGTTAAGTCTTGTGAAAAT |
| | sh3 | CATACAAGAACATGAGATTTC |

**Supplementary Table S2: Primary antibodies used for immunohistochemistry and western blot**

| Antibodies | Concentration | Specificity | Company |
|---|---|---|---|
| PTP4A1 | 1:100 IHC<br>1:1000 WB | Rabbit polyclonal | SIGMA-ALDRICH |
| β-actin | 1:1000 WB | Mouse monoclonal | Abcam |
| PI3K | 1:1000 WB | Rabbit monoclonal | Cell Signaling Technology |
| p-PI3K(Tyr458/Tyr199) | 1:1000 WB | Rabbit monoclonal | Cell Signaling Technology |
| Akt | 1:1000 WB | Rabbit monoclonal | Cell Signaling Technology |
| p-Akt(Thr308) | 1:1000 WB | Rabbit monoclonal | Cell Signaling Technology |
| p-Akt(Ser473) | 1:1000 WB | Rabbit monoclonal | Cell Signaling Technology |
| GSK-3β | 1:1000 WB | Rabbit monoclonal | Cell Signaling Technology |
| p-GSK-3β(Ser9) | 1:1000 WB | Rabbit monoclonal | Cell Signaling Technology |
| CyclinD1 | 1:1000 WB | Rabbit monoclonal | Cell Signaling Technology |
| E-cadherin | 1:1000 WB | mouse monoclonal | Abcam |
| N-cadherin | 1:1000 WB | Rabbit polyclonal | Abcam |
| Vimentin | 1:1000 WB | Rabbit monoclonal | Abcam |
| α-SMA | 1:1000 WB | Rabbit polyclonal | Abcam |
| Zeb1 | 1:1000 WB | Rabbit monoclonal | Cell Signaling Technology |
| Slug | 1:1000 WB | Rabbit polyclonal | Abcam |
| Snail | 1:1000 WB | Rabbit monoclonal | Cell Signaling Technology |

**Supplementary Table S3: Correlation between tumor PTP4A1 expression and clinicopathologic characteristics in ICC (n=322)**

| Characteristics | PTP4A1 | | |
|---|---|---|---|
| | **Low** | **High** | ***P*** |
| Age, years | | | |
| ≤51 | 57 | 112 | 0.485 |
| > 51 | 58 | 95 | |
| Gender | | | |
| Female | 51 | 77 | 0.235 |
| Male | 64 | 130 | |
| Hepatitis history | | | |
| No | 75 | 123 | 0.340 |
| Yes | 40 | 84 | |
| CA199 (U/ml) | | | |
| ≤36 | 61 | 96 | 0.295 |
| >36 | 54 | 111 | |
| Liver cirrhosis | | | |
| No | 90 | 146 | 0.149 |
| Yes | 25 | 61 | |
| Tumor size (cm) | | | |
| ≤5 | 62 | 83 | **0.020** |
| > 5 | 53 | 124 | |
| Tumor number | | | |
| Single | 92 | 152 | 0.222 |
| Multiple | 23 | 55 | |
| Lymph node metastasis | | | |
| No | 105 | 161 | **0.002** |
| Yes | 10 | 46 | |
| Vascular invasion | | | |
| No | 101 | 175 | 0.507 |
| Yes | 14 | 32 | |
| Tumor differentiation | | | |
| I-II | 85 | 164 | 0.331 |
| III-IV | 30 | 43 | |
| TNM stage | | | |
| I +II | 99 | 149 | **0.004** |
| III+IV | 16 | 58 | |

**Abbreviations:** TNM, tumor-nodes-metastases.
* *P* value < 0.05 was considered statistically significant. The Pearson Chi-square test was used.

**Supplementary Table S4: Univariate and multivariate analysis of factors associated with recurrence and survival in ICC (n=322)**

| Variables | OS | | | | TTR | | | |
|---|---|---|---|---|---|---|---|---|
| | Univariate | Multivariate | | | Univariate | Multivariate | | |
| | *P* | HR | 95%CI | *P* | *P* | HR | 95%CI | *P* |
| Age, years (>51vs.≤51) | 0.338 | | | NA | 0.519 | | | NA |
| Gender (male vs. female) | 0.495 | | | NA | 0.736 | | | NA |
| Hepatitis history (yes vs. no) | 0.057 | | | NA | 0.310 | | | NA |
| CA199 (U/ml) (>36 vs. ≤36) | **0.033** | | | NS | 0.061 | | | NA |
| Liver cirrhosis (yes vs. no) | 0.275 | | | NA | 0.170 | | | NA |
| Tumor size (cm) (>5 vs. ≤5) | 0.203 | | | NA | 0.132 | | | NA |
| Tumor multiplicity (multiple vs. single) | **0.034** | **1.458** | **1.037-2.050** | **0.030** | **0.000** | **1.707** | **1.230-2.370** | **0.001** |
| Lymph node metastasis (yes vs. no) | **0.000** | **1.608** | **1.018-2.540** | **0.042** | **0.000** | **2.443** | **1.524-3.916** | **0.000** |
| Vascular invasion (yes vs. no) | 0.221 | | | NA | **0.015** | | | NS |
| Tumor differentiation (poor vs. well) | 0.201 | | | NA | 0.501 | | | NA |
| TNM stage (III-II vs. I) | **0.004** | | | NS | **0.006** | | | NS |
| PTP4A1tumor (High vs. Low) | **0.005** | **1.479** | **1.063-2.056** | **0.020** | **0.001** | **1.587** | **1.149-2.192** | **0.005** |

Univariate analysis was calculated by the Kaplan–Meier method (log-rank test). Multivariate analysis was done using the Cox multivariate proportional hazard regression model with stepwise manner.
OS, overall survival; TTR, time to recurrence; TNM, tumor-nodes-metastases; HR, hazard ratio; CI, confidential interval; NA, not adopted; NS, not significant.
